# Targeted expression of the arsenate reductase HAC1 identifies cell type specificity of arsenic metabolism and transport in plant roots

**DOI:** 10.1093/jxb/eraa465

**Published:** 2020-10-10

**Authors:** Sina Fischer, Eduardo Sánchez-Bermejo, Xuejie Xu, Paulina Flis, Priya Ramakrishna, Mary Lou Guerinot, Fang-Jie Zhao, David E Salt

**Affiliations:** 1 Future Food Beacon of Excellence and the School of Biosciences, University of Nottingham, Nottingham, UK; 2 State Key Laboratory of Crop Genetics and Germplasm Enhancement, College of Resources and Environmental Sciences, Nanjing Agricultural University, Nanjing, China; 3 Department of Biological Sciences, Dartmouth College, Hanover, NH, USA; 4 Biology Center of the Czech Academy of Sciences, Czech Republic

**Keywords:** Accumulation, arsenate, arsenate reductase, export, speciation, tissue-specific expression, tolerance

## Abstract

High Arsenic Concentration 1 (HAC1), an *Arabidopsis thaliana* arsenate reductase, plays a key role in arsenate [As(V)] tolerance. Through conversion of As(V) to arsenite [As(III)], HAC1 enables As(III) export from roots, and restricts translocation of As(V) to shoots. To probe the ability of different root tissues to detoxify As(III) produced by HAC1, we generated *A. thaliana* lines expressing *HAC1* in different cell types. We investigated the As(V) tolerance phenotypes: root growth, As(III) efflux, As translocation, and As chemical speciation. We showed that HAC1 can function in the outer tissues of the root (epidermis, cortex, and endodermis) to confer As(V) tolerance, As(III) efflux, and limit As accumulation in shoots. HAC1 is less effective in the stele at conferring As(V) tolerance phenotypes. The exception is HAC1 activity in the protoxylem, which we found to be sufficient to restrict As translocation, but not to confer As(V) tolerance. In conclusion, we describe cell type-specific functions of HAC1 that spatially separate the control of As(V) tolerance and As translocation. Further, we identify a key function of protoxylem cells in As(V) translocation, consistent with the model where endodermal passage cells, above protoxylem pericycle cells, form a ‘funnel’ loading nutrients and potentially toxic elements into the vasculature.

## Teaser

In roots we uncover cell type-specific functions of the arsenate reductase HAC1 spatially separating control of arsenic tolerance and translocation, and highlight the protoxylem in export of arsenic to shoots.

## Introduction

Arsenic (As) is a potentially toxic trace element that occurs naturally in the Earth’s crust. For human populations not exposed to high As in drinking water, As intake from foodstuffs represents the most important route of As exposure (Zhao *et al.*, 2010). A large proportion of the dietary intake of As comes from rice and rice-based products, although fruit and vegetables, such as apples, potato, carrots, pulses, and spinach, can also be sources of As (Creger and Peryea, 1992; Larsen *et al.*, 1992; Schoof *et al.*, 1999; Meharg *et al.*, 2009). In order to minimize dietary intake of As, a greater understanding of its accumulation in plant-based foods is required.

Arsenic is taken up and transported within plants as both arsenate [As(V)] and arsenite [As(III)] via phosphate transporters and aquaporins, respectively (Abedin *et al.*, 2002; Mudge *et al.*, 2002; Ma *et al.*, 2008; Kamiya *et al.*, 2009; Wang *et al.*, 2016). As(V) is chemically reduced to As(III) in plants (Pickering *et al.*, 2000), suggesting the involvement of an arsenate reductase enzyme. In the search for the plant arsenate reductase, various homologues of the *Saccharomyces cerevisiae* (yeast) arsenate reductase (ScACR2) have been identified and their As(V) reductase activities assayed. The *Arabidopsis thaliana* homologue *AtACR2* was shown to affect As(V) tolerance, to complement the *Escherichia coli arsenate reductase* (*arsC*) deletion mutant, and to have As(V) reductase activity when expressed in an *in vitro* system (Bleeker *et al.*, 2006; Dhankher *et al.*, 2006). The *Oryza sativa* (rice) homologues *OsACR2;1* and *OsACR2;2* exhibited As(V) reductase activity when expressed in an *in vitro* system (Duan *et al.*, 2007) as well as the *Pteris vittata* homologue *PvACR3* (Ellis *et al.*, 2006). While all these studies attributed As(V) reductase activity to these homologues based on findings in heterologous systems, *in vivo* evidence was lacking until it was established that *AtACR2* has no impact on As(V) reduction *in vivo* (Liu *et al.*, 2012).

After a decade-long search (Salt, 2017), the gene for As(V) reduction in plants was described in two independent studies. One quantitative trait locus (QTL) study mapped At2g21045, a rhodanase family member, as the causal gene for As(V) tolerance in *A. thaliana* (Sánchez-Bermejo *et al.*, 2014). A knockout of the gene caused As(V), but not As(III), hypersensitivity, and arsenate reductase activity was established *in vivo* and *in vitro*. The second study mapped the same gene *High Arsenic Content 1* (*HAC1*) in a genome-wide association (GWA) mapping study for leaf As accumulation in *A. thaliana* (Chao *et al.*, 2014). The T-DNA knockout line *hac1-1* showed an elevated leaf As concentration. Arsenate reductase activity was also shown both *in vivo* and *in vitro*. HAC1 is an arsenate reductase, located in the root epidermis, where it converts As(V) into As(III) (Chao *et al.*, 2014). Additionally, the protein was shown to be required for As(III) efflux from roots exposed to As(V). The authors documented that despite As(III) contents comparable with those in roots of wild-type plants, As(III) export from *hac1-1* plants was much reduced in comparison with the wild type. This suggests that HAC1 has a specific role in the reduction of As(V) to generate As(III) for efflux (Chao *et al.*, 2014; Wang *et al.*, 2018). These discoveries in *A. thaliana* have also been extended to rice. In rice, three HAC1 arsenate reductases (OsHAC1;1, OsHAC1;2, and OsHAC4) were found to play important roles in As(V) reduction in roots, and in limiting As accumulation in shoots (Shi *et al.*, 2016; Xu *et al.*, 2017).

Though the direct function of the HAC1 proteins as arsenate reductases is now established, the mechanisms whereby they function to achieve As(III) export and avoid high shoot As concentrations remain to be unravelled. Does HAC1 capture incoming As(V) in the epidermis and interact with specific As(III) exporters at the plasma membrane to achieve As(III) export? Could HAC1 function in other cell layers of the root, and not be strictly dependent on an epidermally localized As(III) export system?

To investigate these questions, plants were generated which expressed *HAC1* in a cell type-specific manner in the null *hac1-1* mutant background. This ensured that HAC1 would be present in different root tissues such as the epidermis, as in wild-type plants, the cortex, endodermis, or different stele tissues. This was achieved by placing the *HAC1* gene under control of promoters known to be only active in specific root tissues (Imlau *et al.*, 1999; Birnbaum *et al.*, 2003; Abas *et al.*, 2006; Lee *et al.*, 2006; Jaillais *et al.*, 2007; Mustroph *et al.*, 2009; Marquès-Bueno *et al.*, 2016). In order to confirm the presence of HAC1 in the intended root tissue the protein was tagged with mCherry or green fluorescent protein (GFP). Both fluorescent tags allow the verification of the localization of HAC1 using confocal microscopy. We document the effects of this cell type-specific expression of *HAC1* on As(V) tolerance, As translocation, As(III) efflux, and As speciation.

## Materials and methods

### Plant materials and growth conditions

All *A. thaliana* transgenic lines were generated in the *hac1-1* genetic background (Chao *et al.*, 2014; Sánchez-Bermejo *et al.*, 2014). The MultiSite Gateway system was chosen to clone the selected promoters and the *HAC1* ORF. The plasmids pDONR-P4-P1R and pDONR221 were used as donor plasmids for this first step. The attB4 and attB1r Gateway sites were added to either end of the promoter PCR product, while the sites attB1 and attB2 were added to the ends of the *HAC1* ORF (for cloning primers including recombination sites, see Supplementary Table S1 at *JXB* online). In two subsequent independent BP clonase reactions, the entry clones pDONOR-P4-R1R-promoter and pDONOR221-*HAC1* were generated. These two entry clones, along with the selected tag entry clone [mCherry (pEN-R2-L3) and GFP (pEN-R2-L3-GSgreen)], were used in a final LR reaction, using the destination vector R4-R3 (pH7m34GW), to generate the promoter–*HAC1* ORF-tag fusion construct. For transformations, we used chemically competent cells of the *Escherichia coli* DH5α strain, and for transformation of *A. thaliana hac1-1* we used electrocompetent *Agrobacterium tumefaciens* cells of the strain GV3101. Plants were transformed using the floral dip method (Zhang *et al.*, 2006). The final set of transgenic lines were selected by PCR, and by identifying the fluorescent signal of the mCherry or GFP reporter proteins by confocal microscopy.

**Fig. 1.**
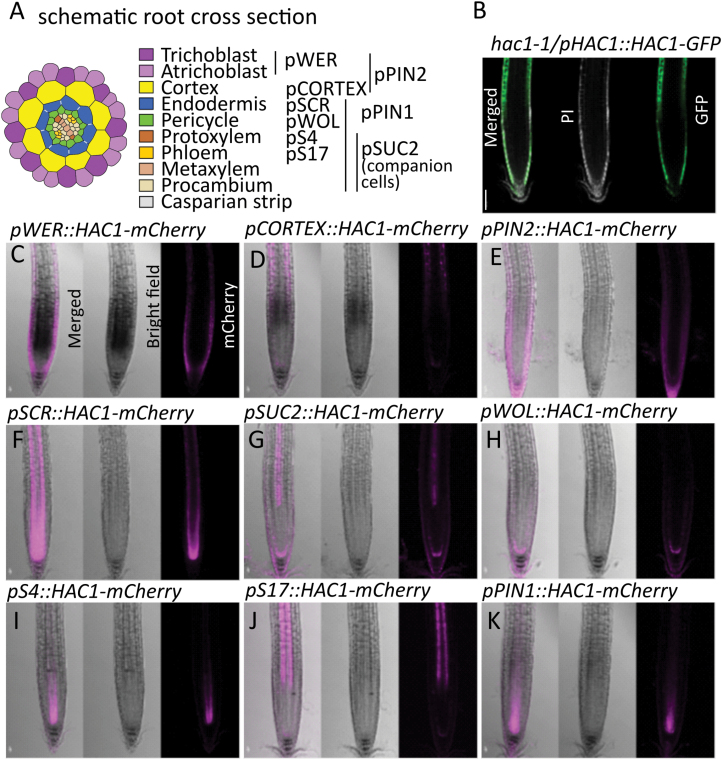
Localization of HAC1. (A) Schematic radial root cross-section of A. *thaliana* with the different promoters used to induce tissue-specific expression of *HAC1.* (B–K) Representative confocal images of A. *thaliana* roots in longitudinal view that show HAC1 localization under the control of its native promoter with a GFP tag (B), and the tissue-specific promoters with an mCherry tag (C–K). A plant with a GFP fusion protein (B) was stained with propidium iodide (PI) to show cell boundaries, while for lines (C–K) expressing the gene with an mCherry tag, the bright field images help visualize cell boundaries. All constructs are in the *hac1-1* background, Scale bar for all=100 µm.

In order to confirm the localization of HAC1 in roots, plants were grown on 1/2 strength Murashige and Skoog (MS) medium [2.1 g l^–1^ MS (M5519, Sigma), 2.5 mM MES, 1% (w/v) sucrose, 1% (w/v) agar Type A, pH 5.7] for 5 d in long-day conditions (16 h:8 h, light:dark, 139–169 m^–2^ s^–1^ at 23 °C) for confocal imaging. The fluorescent reporter lines were imaged using a confocal laser microscope (Leica TCS SP5). Lines expressing the *GFP* tag were stained with 1.2% propidium iodide solution for 2 min and subsequently rinsed with water before imaging. Plants expressing *mCherry*-tagged *HAC1* were imaged without any staining. GFP and mCherry were excited at 488 nm and 514 nm, respectively, and collected in the emission range 500–550 nm and 550–650 nm, respectively. Images were analysed using ImageJ 1.52. Lines displayed in Fig. 1 are *hac1-1/pHAC1::HAC1-GFP#6-1-9*, *pWER::HAC1-mCherry#50*, *pCORTEX::HAC1-mCherry#39*, *pPIN2::HAC1-mCherry#9.6*, *pSCR::HAC1-mCherry#6*, *pSUC2::HAC1-mCherry#1.3*, *pWOL::HAC1-mCherry#8.4*, *pS4::HAC1-mCherry#15.4*, *pS17::HAC1-mCherry#2.2*, and *pPIN1::HAC1-mCherry#12.8* and are representative for different insertions of the same transgene. In order to assess plant tolerance to As(V), the root length was measured in plants grown on media containing 1/2 strength MS for 7 d in long-day conditions (16 h light at 23 °C), with or without the addition of 200 µM AsO_4_Na_2_H, a concentration based on previous experiments (Chao *et al.*, 2014).

Shoot As contents were also analysed in plants grown in an artificial soil. Plants were grown for 5 weeks in a climate-controlled growth room with short-day conditions (10 h:14 h, light:dark, 19–21 °C, 70–80% humidity). Peat Jiffy® pellets (Jiffy Products International BV, Zwijndrecht, The Netherlands) were soaked in a solution (104 pellets in 2.6 litres) containing 0.142 µM Na_2_HAsO_4_·7H_2_O, providing a final concentration in dry soil of 5.6 ppm As. Each fully expanded Jiffy® peat pellet is ~3 cm in diameter and 4 cm high. Seeds were sown directly onto soil and stratified for 2 d at 4–6 °C in the dark. Subsequently, plant growth trays were moved to the growth room and watered once per week with 1/4 strength Hoagland solution (250 µM NH_4_H_2_PO_4_, 500 µM MgSO_4_, 700 µM CaNO_3_, 1.5 mM KNO_3_, 12.5 µM Fe-HBED, 4.63 µM H_3_BO_3_, 32 nM CuSO_4_, 0.915 µM MnCl_2_, 77 nM ZnSO_4_, 11 nM MoO_3_, pH 5.7) (1 litre per tray containing 104 pellets). After 1 week, seedlings were removed to leave a single individual plant per pellet, and pellets were randomized within the tray.

For As uptake, efflux, and speciation, *A. thaliana* lines were grown hydroponically with 1/5 strength Hoagland nutrient solution (Wang *et al.*, 2018). Thirty-day-old plants were exposed to 5 µM As(V) for 24 h, with four replicates for each line. This concentration is subtoxic and suitable for speciation and efflux experiments as used in previous studies (Liu *et al.*, 2010; Chao *et al.*, 2014). Phosphate was withheld from the nutrient solution to facilitate As(V) uptake. As(V) uptake and As(III) efflux were estimated by measuring the changes in As speciation in the nutrient solution, as described previously (Wang *et al.*, 2018). At the end of As(V) exposure, roots were desorbed of apoplastic As in an ice-cold solution containing 1 mM K_2_HPO_4_, 0.5 mM Ca(NO_3_)_2_, and 5 mM MES (pH 5.5) for 10 min (Liu *et al.*, 2010). Roots and shoots were rinsed with deionized water, blotted dry, and weighed. Plant samples were ground in liquid nitrogen to a fine powder. Subsamples (~0.1 g) of the ground materials were extracted with 10 ml of a phosphate buffer solution (2 mM NaH_2_PO_4_, 0.2 mM Na_2_-EDTA, pH 5.5). The As species in the nutrient solution, and in root and shoot extracts, were determined on the day of the harvesting to avoid degradations, using HPLC linked to inductively coupled plasma MS (HPLC-ICP-mass spectrometry; NexIon 300x, Perkin-Elmer) (Liu *et al.*, 2010).

### Elemental analysis

Leaf elemental content was measured using ICP-MS as previously described (Danku *et al.*, 2013). Briefly, harvested leaves were washed with 18.2 MΩ cm Milli-Q Direct water (Merck Millipore), placed in Pyrex test tubes (16×100 mm), and dried at 88 °C for 24 h. After cooling, eight of 108 samples from each sample set were weighed. After weighing the appropriate number of samples (these dry weights were used to calculate the dry weights of the rest of the samples; for details, see Danku *et al.*, 2013), all samples, including four blank controls, were digested with 1 ml of concentrated trace metal grade nitric acid Primar Plus (Fisher Chemicals) containing an indium internal standard, in dry block heaters (SCP Science; QMX Laboratories) at 115 °C for 4 h. After cooling, digested samples were diluted to 10 ml with 18.2 MΩ cm Milli-Q Direct water (Merck Millipore) and subsequently analysed using ICP-MS (PerkinElmer NexION 2000 equipped with Elemental Scientific Inc. autosampler), in the collision mode (He). A matrix-matched liquid reference material composed of pooled samples was prepared before the beginning of the sample run and used every ninth sample to correct for variation within ICP-MS analysis runs. The calibration standards were prepared from solutions of single element standards (Inorganic Ventures; Essex Scientific Laboratory Supplies Ltd, Essex, UK). Sample concentrations were calculated using external calibration methods within the instrument software. The final elemental concentrations were obtained by normalizing the element concentrations to the sample dry weights. These were calculated using the dry weight of previously weighed samples and their solution concentrations based on a heuristic algorithm which uses the best-measured elements in these samples, described in detail at www.ionomicshub.org/ and by Lahner *et al.*, (2003).

### Root cross-section analysis

Images of root cross-sections were gathered from three publications (Hawker and Bowman, 2004; Sutka *et al.*, 2011; Dyson *et al.*, 2014), imported into ImageJ 1.52, and each tissue layer area was measured using the hand-trace tool. The area, in pixels, of each layer was normalized to the overall root cross-section area to gather the contribution of the respective tissue to the overall size of the root. An average of the 13 analysed images was calculated. The data are displayed in Supplementary Table S2.

### Statistical analysis

Significant differences between genotypes were determined by one-way ANOVA utilizing R [R studio version 1.2.1335, R version 3.6.0 (2019-04-26)], including packages bestNormalize version 1.5.0, emmeans version 1.4.6, sjPlot version 2.8.3, multcomp version 1.4-13, plyr version 1.8.6, Rmisc version 1.5, and ggplot2 version 3.3.0. Boxplots were used to visualize data with sample sizes of ≥5, while bar plots were used when sample sizes were <5 (Krzywinski and Altman, 2014).

## Results

### Cell-type specific localization of *HAC1*

In order to assess the effect of *HAC1* expression on various phenotypes related to As(V) tolerance, we used different promoters to express *HAC1* in a cell type-specific manner (Fig. 1A). *HAC1* was fused in-frame with a fluorescent *GFP* or *mCherry* tag. No native *HAC1* expression was present in these lines as they were transformed into the *hac1-1* T-DNA insertion mutant background.

The native promoter of *HAC1* was used to drive expression of *GFP*-tagged *HAC1*. The promoter is known to be active in the epidermis (Chao *et al.*, 2014) along the full length of the root and lateral root cap (Fig. 1B; Supplementary Fig. S1). The pattern does not change upon As(V) exposure (Supplementary Fig. S1). *HAC1* has also previously been reported to be expressed in the pericycle (Chao *et al.*, 2014), though we were unable to detected pericycle expression in the present study.

For expression of *HAC1* in other cell types. the localization of HAC1–mCherry was confirmed by confocal microscopy (Fig. 1C–K). The mCherry signal was detectable in all the lines in the expected root cell types. The *WEREWOLF* (*pWER*, AT5G14750) promoter leads to mCherry signal in the epidermis, in both atrichoblasts and trichoblasts at the root tip and the lateral root cap, but is only active in the atrichoblasts in more mature root (Marquès-Bueno *et al.*, 2016). Consequently, mCherry signal in these lines was detectable in the epidermis of the root tip and the root hair cells. The promoter *Cortex* (*pCortex*, AT3G05150) lines showed mCherry signal in the cortex, starting in the elongation zone, as previously observed (Lee *et al.*, 2006). The *PIN2* (*pPIN2*, At5g57090) promoter leads to mCherry signal in the epidermis and cortex (Abas *et al.*, 2006; Marquès-Bueno *et al.*, 2016). The *SCARECROW* promoter (*pSCR*, AT3G54220) leads to mCherry signal in the endodermis (Birnbaum *et al.*, 2003; Mustroph *et al.*, 2009), while the *SUC2* promoter of the *sucrose-proton symporter 2* (*pSUC2*, AT1G22710) leads to mCherry signal in the phloem companion cells (Imlau *et al.*, 1999; Marquès-Bueno *et al.*, 2016). The *WOODEN LEG* (*pWOL*, AT2G01830) promoter leads to mCherry signal in the pericycle (Birnbaum *et al.*, 2003; Mustroph *et al.*, 2009), with the greatest intensity above the quiescent centre. The *S4* (*pS4*, AT3G25710) promoter leads to mCherry signal in the protoxylem in the meristematic zone, while the *S17* (*pS17*, AT2G22850) promoter leads to mCherry signal in the stele, and more precisely the phloem of the early elongation zone (Lee *et al.*, 2006; Marquès-Bueno *et al.*, 2016). Promoter *PIN1* (*pPIN1*, AT1G73590) lines showed mCherry signal in the stele as previously reported for *pPIN1:PIN1-GFP* (Jaillais *et al.*, 2007; Marquès-Bueno *et al.*, 2016).

### Arsenate tolerance

**Fig. 2.**
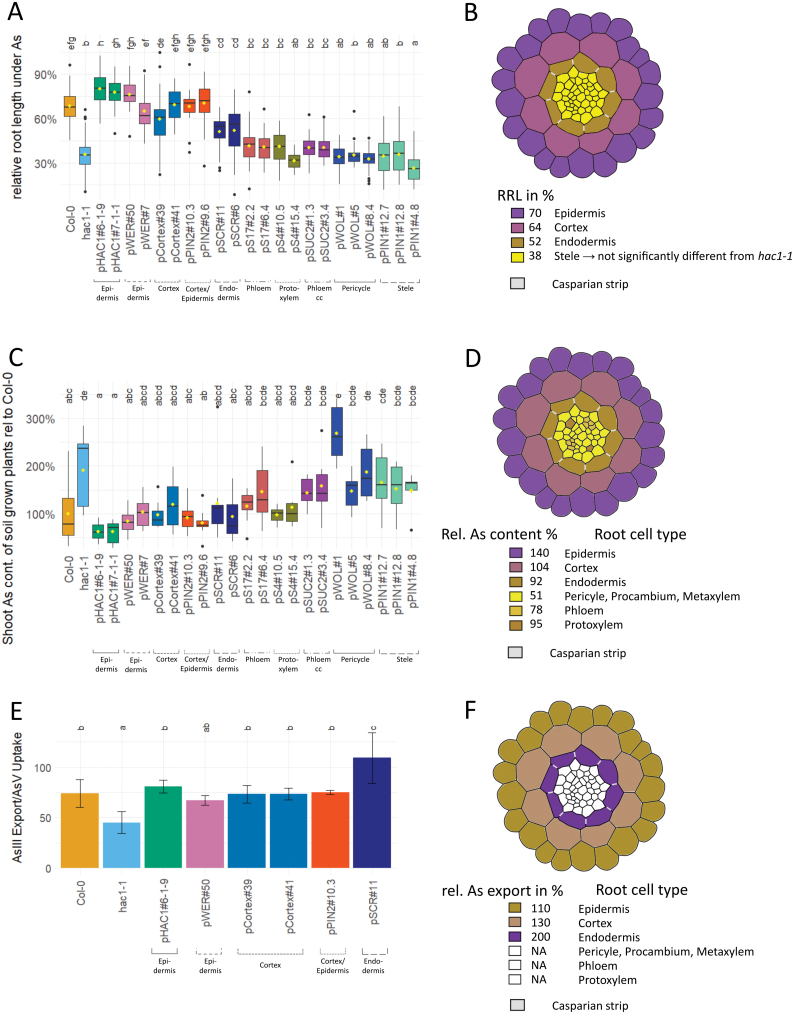
Tolerance, As accumulation in shoots, and As export in response to AsO_4_Na_2_H stress in *A. thaliana*. (A and B) Tolerance. Plants were grown under control conditions and 200 µM AsO_4_Na_2_H stress to assess the relative root length. Plants expressing *HAC1* under control of tissue-specific promoters in the *hac1-1* background were compared with the wild type Col-0 and the *hac1-1* mutant. (A) Root length normalized to the root length under control conditions. *n*=25–204. (B) A schematic of a root cross-section. Colouring is dependent on As tolerance achieved by expression of *HAC1* in the respective tissue, with purple showing high and brown low tolerance. Yellow shows the relative root length (RRL) not significantly different from *hac1-1*. (C and D) Accumulation. Plants were grown on soil supplemented with low levels of As (148 nmol/104 Jiffy (R) pellets) and shoot As contents were determined. (C) Shoot As levels relative to As levels in Col-0. *n*=7–10. (D) Schematic of a root cross-section. Rel. As content is calculated as As content in Col-0/As content in lines expressing HAC1 in the epidermis, cortex, endodermis, pericycle, phloem, and protoxylem, respectively. Colouring is dependent on rel. As content, with purple showing high and yellow low rescue of the wild-type phenotype. (E and F) Export. Plants were grown hydroponically and, after 24 h of treatment with 5 µM As(V) and As(III), export was assessed. (E) Relative As(III) export normalized to As(V) uptake. As(III) was measured in buffer containing roots previously subjected to As(V) to achieve As uptake. Data for uptake and export are displayed in Supplementary Fig. S3. (F) A schematic of a root cross-section. Colouring is dependent on As export achieved by expression of *HAC1* in the respective tissue, with purple showing high and yellow low As export. White: no data. Box plots show the median±1.58×IQR/sqrt(*n*), with outliers in black and the mean in yellow. Bar plots show the mean ±SD. *n*=3. Significant differences are shown with different letters above boxes, the result of a one-way ANOVA with post-hoc Tukey test.

For each line, As(V) tolerance was assessed as growth in the presence of As(V) normalized to growth under control conditions in the absence of added As(V) (Fig. 2A). Under control conditions, roots reached on average a length of 2.7±0.7 cm (Supplementary Fig. S2). The relative root length (RRL) was calculated as growth under As(V) stress/growth under control conditions as a percentage. Growth in the presence of As(V) revealed significant differences among lines (Fig. 2A). In response to As(V), growth of wild-type and *hac1-1* roots was significantly different. Wild-type roots were reduced to 67±11%, while *hac1-1* was reduced to 37±10%. This phenotype, which is related to the loss of *HAC1* in the mutant line, was fully rescued by *HAC1-GFP* expressed under its native promoter in two independent lines. Full rescue of As(V) tolerance in the *hac1-1* mutant background back to wild-type levels was also achieved in lines expressing *HAC1-mCherry* from the *Cortex* promoter, the *PIN2* promoter, and the *WER* promoter. Each of these lines was not significantly different from the wild type. However, lines expressing *HAC1-mCherry* from the *SCR* promoter showed a significant but only partial rescue of the As(V) sensitivity of *hac1-1*, to 78% of the As(V) tolerance of the wild type. Expression of *HAC1-mCherry* from the promoters *PIN1*, *WOL*, *SUC2*, *S4*, and *S17* did not show rescue of the *hac1-1* As(V) sensitivity, and these lines were not significantly different from *hac1-1*. A schematic root cross-section illustrates the average As(V) tolerance achieved by expression of *HAC1* in specific cell types (Fig. 2B), highlighting the role of the outer root tissues for As(V) tolerance.

### Shoot arsenic accumulation

Loss of function of *HAC1* has previously been shown to cause elevated As accumulation in shoots (Chao *et al.*, 2014; Wang *et al.*, 2018). In order to establish in which cell types of the root *HAC1* can function to limit this shoot overaccumulation of As, we investigated shoot As accumulation in our cell type-specific *HAC1* expression lines. Plants were grown on artificial soil to which a subtoxic concentration of As (5.62 mg kg^–1^ As) was added. After 5 weeks of growth, leaves were harvested and As concentration determined on a leaf dry weight basis. The *hac1-1* mutant had double the As concentration compared with the wild type (Fig. 2C), as expected. This As overaccumulation phenotype was fully rescued by expression of *HAC1-GFP* under the control of the *HAC1* native promoter in two independent lines, which showed no significant difference in shoot As from the wild type. Expression of *HAC1* tagged with *mCherry* under control of the *Cortex*, *PIN2*, *S4*, *SCR*, and *WER* promoters also achieved full rescue of wild-type levels of shoot As accumulation, showing no significant difference from the wild type. In contrast, *HAC1-mCherry* expressed from the promoters *S17*, *SUC2*, *PIN1*, and *WOL* was unable to rescue the shoot As overaccumulation of the *hac1-1* mutant, with shoot As levels not significantly different from those of *hac1-1*.

A schematic root cross-section (Fig. 2D) illustrates a similar pattern to that observed for *HAC1* function in As(V) tolerance (Fig. 2B). In this case, a reciprocal colour scheme to that used for As tolerance is used in order to be able to directly contrast high tolerance and low As content. This schematic also highlights the protoxylem cells in the stele, illustrating the effect on shoot As accumulation, but not tolerance, resulting from *HAC1* expression in this cell type.

### Arsenic efflux

In a hydroponic experiment using a subset of our *HAC1* cell type-specific expression lines. As(III) efflux was observed (Fig. 2E). Plants were grown hydroponically for 4 weeks and subsequently the hydroponic solution was supplemented with 5 µM As(V) for 24 h (Supplementary Fig. S3). Differences in growth were observed between different lines, including reduced growth of the *Cortex* and *SCR* promoter lines, and increased growth of the *WER* promoter lines (Supplementary Fig. S3b, c). No visible effects of As(V) treatment were detected (Supplementary Fig. S3a). As observed previously, efflux of As(III) was significantly reduced in *hac1-1* (Chao *et al.*, 2014; Wang *et al.*, 2018). Efflux dropped from 74±14% in the wild type to 45±11% in *hac1-1* when expressed as the ratio of As(III) export to As(V) uptake (Fig. 2E). A similar pattern was also observed in the absolute amount of As(III) export (Supplementary Fig. S3e). Complementation with *HAC1-GFP* under control of its native promoter re-established As(III) export to wild-type levels (Fig. 2E; Supplementary Fig. S3e). Likewise, *HAC1-mCherry* expression in the promoter lines *Cortex* and *PIN2* resulted in export of As(III) resembling that of the wild type. Even higher amounts of As(III) were exported in plants expressing *HAC1-mCherry* under control of the *SCR* promoter compared with the wild type. In plants where *HAC1-mCherry* was expressed under control of *pWER*, a partial rescue of As(III) export could be observed. A schematic root cross-section (Fig. 2F) illustrates that all outer root tissues and the endodermis are able to facilitate As efflux from the root when expressing *HAC1* in a null *hac1-1* background.

### Arsenic speciation

**Fig. 3.**
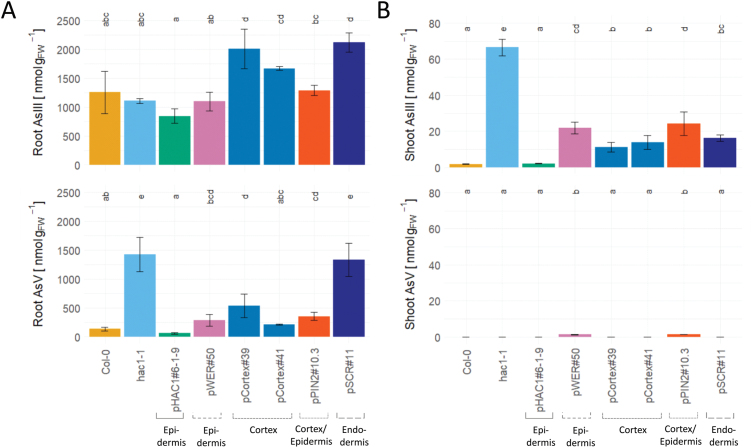
Arsenic speciation in hydroponically grown *A. thaliana* plants. (A) Root and (B) shoot arsenic content were quantified. Values show the total root and shoot As content, respectively, while tissue-specific expression reveals the tissue-specific effect. Bar plots show the mean ±SD. *n*=3. Significant differences are shown with different letters above boxes, the result of a one-way ANOVA with post-hoc Tukey test.

Root and shoot arsenic speciation analysis revealed the distribution of As(III) and As(V) in different tissues after hydroponic growth with a subtoxic concentration (5 µM) of As(V) for 24 h. In roots of the wild type, most (90%) of the As was present as As(III), with only a small portion of As(V) detectable (Fig. 3A). The concentration of As(V) was greatly increased in the root of the *hac1-1* mutant, consistent with HAC1 being an arsenate reductase. As(III) levels in *hac1-1* were similar to those of the wild type. Complementing *hac1-1* with *HAC1-GFP* under the control of the *HAC1* native promoter reduced the amount of As(V) in roots to below wild-type levels. Complementation of *hac1-1* with *HAC1-mCherry* in the epidermis and cortex (pWER, pCortex, and pPIN2) significantly reduced the amount of As(V) in roots. Intriguingly, expression of *HAC1* in the endodermis from the p*SCR* promoter did not reduce root As(V) in comparison with *hac1-1*, and levels remained elevated in comparison with those of the wild type, as observed in *hac1-1.* This indicates that the majority of As(V) is located in the epidermis and cortex of the root. This is possibly due to the difference in size and number of the cells of the different root tissues. The epidermal and cortical tissues make up 76±4% of the area of a root cross-section, while the rest of the tissues contribute 22±4% to the total area (Supplementary Table S2). Tissue-specific expression of *HAC1-mCherry* from the *Cortex* and *SCR* promoters also increased the amount of As(III) in roots to above wild-type levels. This goes along with slightly higher As(V) uptake levels which could account for these differences in As(III) (Supplementary Fig. S3). In shoots, hardly any As(V) is detectable, and virtually all As(V) has been reduced to As(III) (Fig. 3B). A small amount of As(V), just above the quantification limit, remained in some of the cell type-specific promoter lines, in each case contributing <6% to the total amount of arsenic in the shoot. As(III) concentrations in the wild type are low, but in *hac1-1* a large increase in shoot As(III) was detected, as previously observed (Chao *et al.*, 2014; Wang *et al.*, 2018). Full complementation of this overaccumulation of As(III) back to wild-type levels was achieved using the native promoter of *HAC1* to express *HAC1-GFP*, while only a partial reduction of As(III) levels was observable in lines expressing *HAC1-mCherry* in a tissue-specific manner.

## Discussion

We investigated the function of HAC1 in a tissue-specific context. Overall, we found that HAC1 functions best when present in the outer cell types of the root (epidermis, cortex, and endodermis), to confer root As(V) tolerance, enable As(III) export, and keep shoot As levels low. This contrasts with expression of *HAC1* in cell types in the stele where it was not effective at conferring root tolerance to As(V) or limiting As accumulation in shoots. The exception to this is the fact that expression of *HAC1* in the protoxylem cells in the stele is capable of limiting translocation of As to the shoot. However, intriguingly, it does not confer As(V) tolerance when expressed at this location.

When the *hac1-1* mutant was complemented with a GFP-tagged version of HAC1 under control of its native promoter, three wild-type phenotypes were fully restored: growth, As accumulation, and As(III) export (Fig. 2A, C, E). This confirms previous observations and establishes that HAC1 is active in the epidermis (Fig. 1; Supplementary Fig. S1) where it reduces As(V) to As(III). Restoration of wild-type levels of As-related phenotypes [As(V) tolerance measured as root growth, As(III) efflux, As(V) reduction, and shoot As accumulation in *hac1-1* plants expressing *HAC1* in the epidermis] is expected, since the native promoter is also active in this cell type. The observation that this suite of As-related phenotypes is restored when *HAC1* is expressed in the cortex and endodermis allowed us to conclude that the detoxification and export machinery for As(III) is present not only in the epidermis but also in the cortex and the endodermis.

It is known that the detoxification machinery, involving the phytochelatin (PC) synthase enzyme PCS1 and the PC–As(III) transporter ABCC1/2, is present throughout the root of *A. thaliana* (Song *et al.*, 2010; Ryu *et al.*, 2019). Export of As(III), on the other hand, is so far not genetically characterized. As(III) can be transported through aquaporins (Kamiya *et al.*, 2009). Nineteen full-length aquaporins are expressed in the root of *A. thaliana* (Quigley *et al.*, 2001) and could contribute to As(III) export. Their tissue-specific localization has not yet been studied and would provide a first indication of which aquaporins may be relevant for As(III) efflux. Another candidate for As(III) export is PIN2 (Ashraf *et al.*, 2019, Preprint), whose localization coincides with the cell types where *HAC1* expression confers As tolerance, supporting its potential function as an As(III) exporter.

Any As that is not exported from the root as As(III), or sequestered in the vacuole as a PC–As(III) complex, becomes available for translocation to the shoot. *PHO1*, a phosphate transporter involved in loading of phosphate into the xylem, has also been implicated in As(V) translocation in *A. thaliana* (Hamburger *et al.*, 2002; Wang *et al.*, 2018). *PHO1* is expressed in the pericycle (Hamburger *et al.*, 2002; Ryu *et al.*, 2019), and the protein is localized in this tissue (Liu *et al.*, 2012). However, expression of *HAC1* in the pericycle using the *pWOL* and *pPIN1* promoters does not restore As(V) tolerance in our experiments, and neither does it reduce the shoot As overaccumulation phenotype of *hac1-1*. This may be due to the differences in expression patterns for *PHO1*, *WOL*, and *PIN1*. While *PHO1* is expressed in older parts of the root, both *WOL* and *PIN1* show expression mostly in the root tip, lateral root tips, and the root to shoot transition zone (Hamburger *et al.*, 2002; Omelyanchuk *et al.*, 2016). Within the stele, other transporters such as PHT1;3 or PHT1;4, which are thought to be localized to the pericycle and the cell layers interior to the pericycle (Mudge *et al.*, 2002), may contribute to As(V) transport in *A. thaliana*. However, they probably play only a minor role since a knockout of *PHO1* blocks most As(V) translocation to the shoot in the *hac1*-1 null mutant background (Wang *et al.*, 2018).

Interestingly, shoot As overaccumulation in *hac1-1* can be rescued by *HAC1* expression in protoxylem cells from the *pS4* promoter. However, As(V) tolerance as measured by root growth was not restored by expressing *HAC1* in the protoxylem. This highlights the importance of the protoxylem for As(V) translocation. The protoxylem is located underneath the pericycle, specifically the xylem pole pericycle cells. In the mature part of the root, non-suberized passage cells within the endodermis are located above xylem pole pericycle cells (Andersen *et al.*, 2018), providing a direct route of access for ions from the outer root tissues to the xylem. This is thought to create a ‘nutrient funnel’ which allows efficient uptake of ions in the mature suberized zone of the root (Andersen *et al.*, 2018). This funnelling effect created by passage cells is thought to be mediated through the cell-specific expression of ion transporters, as has been demonstrated for *PHO1*. *PHO1* is expressed in a gradient in pericycle cells, showing the strongest expression in the xylem pole pericycle cells, and in endodermal cells adjacent to xylem pole pericycle cells, which correspond to the location of passage cells (Hamburger *et al.*, 2002). From these observations, we speculate that As(V) is funnelled into the stele in the mature region of the root at sites of endodermal passage cells, via the action of PHO1. It is PHO1, located in greater amounts in the xylem pole pericycle cells, which loads As(V) into the protoxylem cells. However, the presence of HAC1 in protoxylem cells is sufficient to block further transport of As to the shoot by converting As(V) to As(III). The protoxylem is the first part of the xylem to differentiate, and continues throughout the whole root into the hypocotyl (Keller *et al.*, 1989). *HAC1* expression solely in protoxylem cells is sufficient to block transport of As to the shoot. This strongly supports the earlier conclusion that PHO1 transports As(V) (Wang *et al.*, 2018), and identifies the critical role of protoxylem cells in loading of As(V) into the xylem.

Our results also demonstrate the fact that the endodermis acts as the boundary cell layer between the outer tissues where HAC1 can function to mediate the suite of processes required for As tolerance, and the inner cell types of the stele where HAC1 does not function in As tolerance. This barrier function is enabled by the Casparian strip in endodermal cell walls, forming an interlocking barrier to apoplastic diffusion (Barbosa *et al.*, 2019). In support of this, elevated levels of shoot As have been observed in the *sgn3* mutant which has defective Casparian strips (Pfister *et al.*, 2014; Huang and Salt, 2016). In plants with a defective Casparian strip-based diffusion barrier, As could move freely into and out of the stele. In such plants, *HAC1* might be expected to complement *hac1-1* As-related phenotypes even when expressed in the stele. Future development of lines in which *HAC1* is expressed in a cell type-specific manner in roots of the *hac1 sgn3* double mutant would allow this hypothesis to be tested.

**Fig. 4.**
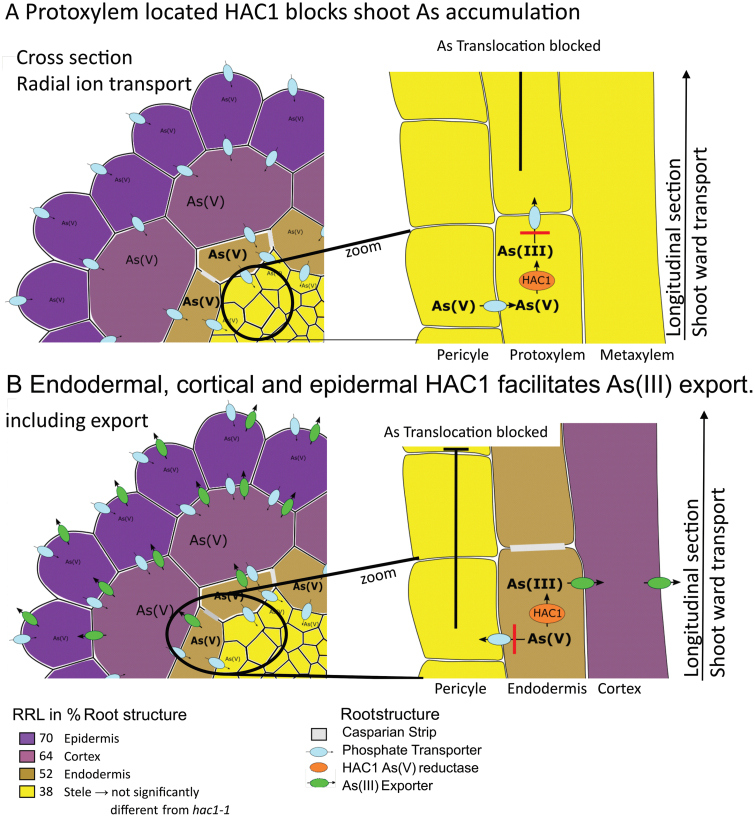
Schematic arsenic transport in *A. thaliana* plants expressing *HAC1* in different tissues. (A) Protoxylem blocks shoot As accumulation. Expression of *HAC1* in protoxylem cells, although not affecting tolerance as assessed through relative root length (RRL), blocks shoot As transport. Phosphate transporters responsible for loading of As(V) into the xylem are not able to translocate As(III), produced by HAC1, within the xylem. Tolerance is conferred by expression of *HAC1* in the outer root tissues, as indicated in the cross-section through a purple colour, while the lighter yellow in the stele indicates As intolerance. (B) Endodermal, cortical, and epidermal HAC1 facilitates As(III) export. Expression of *HAC1* in the endodermis blocks As translocation as well, but additionally also leads to effective As(III) detoxification via export (green membrane protein).

From our observations, we have developed a model (Fig. 4) for As metabolism and transport in roots. Upon uptake of As(V) by PHT1;1 and PHT1;2, As(V) gets reduced in the epidermis by HAC1, producing As(III). A portion of the As(III) produced is exported from the root, via a currently unknown transporter(s), possibly aquaporins or PIN2. This efflux is effective at conferring tolerance to As when active in the outer cells of the root (epidermis, cortex, and endodermis). Another fraction of As(III) is detoxified through binding to PCs and storage in vacuoles. These processes are also effective if HAC1 is present in the epidermis, cortex, or endodermis. As(V) that arrives at endodermal cells is loaded into the protoxylem via PHO1 for translocation to the shoot. The existence of this specific pathway is supported by the observation that HAC1 activity, converting As(V) to As(III) in this specific cell type, can block As(V) translocation.

In summary, we have uncovered critical aspects of As metabolism and transport in *A. thaliana* which move our understanding of these processes to the cellular level. We define new pathways for As detoxification in plants, including the identification of protoxylem cells as part of a critical pathway for As(V) translocation to the shoots. This refined cellular view of As metabolism and transport provides both new and important avenues to understand how plants cope with the toxic effects of As exposure, and also new insights into possible approaches to limit As accumulation in food crops.

## Supplementary data

The following supplementary data are available at *JXB* online.

Fig. S1. Microscopic images of *hac1-1*/*pHAC1::HAC1-GFP* roots showing the localization of HAC1–GFP in plants grown under control conditions or under As(V) stress.

Fig. S2. Boxplot showing the absolute root growth of all lines expressing *HAC1* in a tissue-specific manner as well as the wild types and *hac1-1* under control conditions.

Fig. S3. Image of plants grown hydroponically to assess root As(III) export as well as biomass quantification and As(V) uptake and As(III) export in µg g FW^–1^.

Table S1. List of oligonucleotides used for the generation of tissue-specific lines.

Table S2. Raw data for the quantification of the area of individual tissues in Arabidopsis root cross-sections.

eraa465_suppl_Supplemenatry-Figures-S1-S3_and_Tables-S1-S2

## Data Availability

The data supporting the findings of this study are available from the corresponding author (David E. Salt) upon request.
