# Supplementary material for: Targeted expression of the arsenate reductase HAC1 identifies cell type specificity of arsenic metabolism and transport in plant roots
Source: J Exp Bot. 2020 Oct 10;72(2):415–25. doi: 10.1093/jxb/eraa465 (PMC7853597; doi:10.1093/jxb/eraa465)
Supplement: eraa465_suppl_Supplemenatry-Figures-S1-S3_and_Tables-S1-S2 [file eraa465_suppl_supplemenatry-figures-s1-s3_and_tables-s1-s2.pdf]

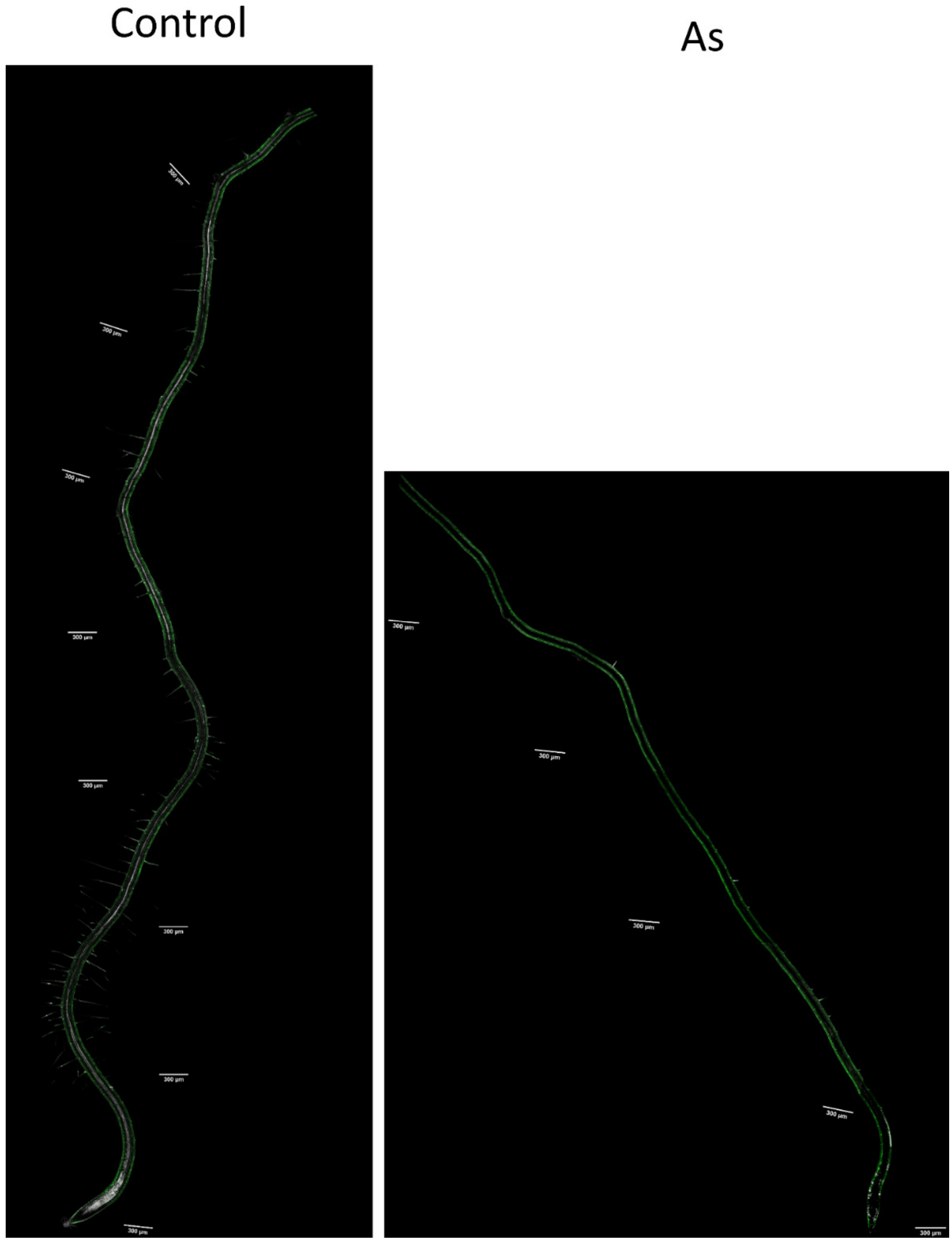

Fig. S1 Localization of HAC1 in hac1-1/pHAC1::HAC1-GFP. Microscopic analysis of localization of HAC1 tagged with GFP in PI stained *hac1-1/pHAC1::HAC1-GFP* lines. Plants were grown under control conditions or under 200µM AsO_4Na_2H. Images were taken along a 7d old root and show epidermal localization of HAC1 throughout the root. Scale bar for all images in white marks 300µM.

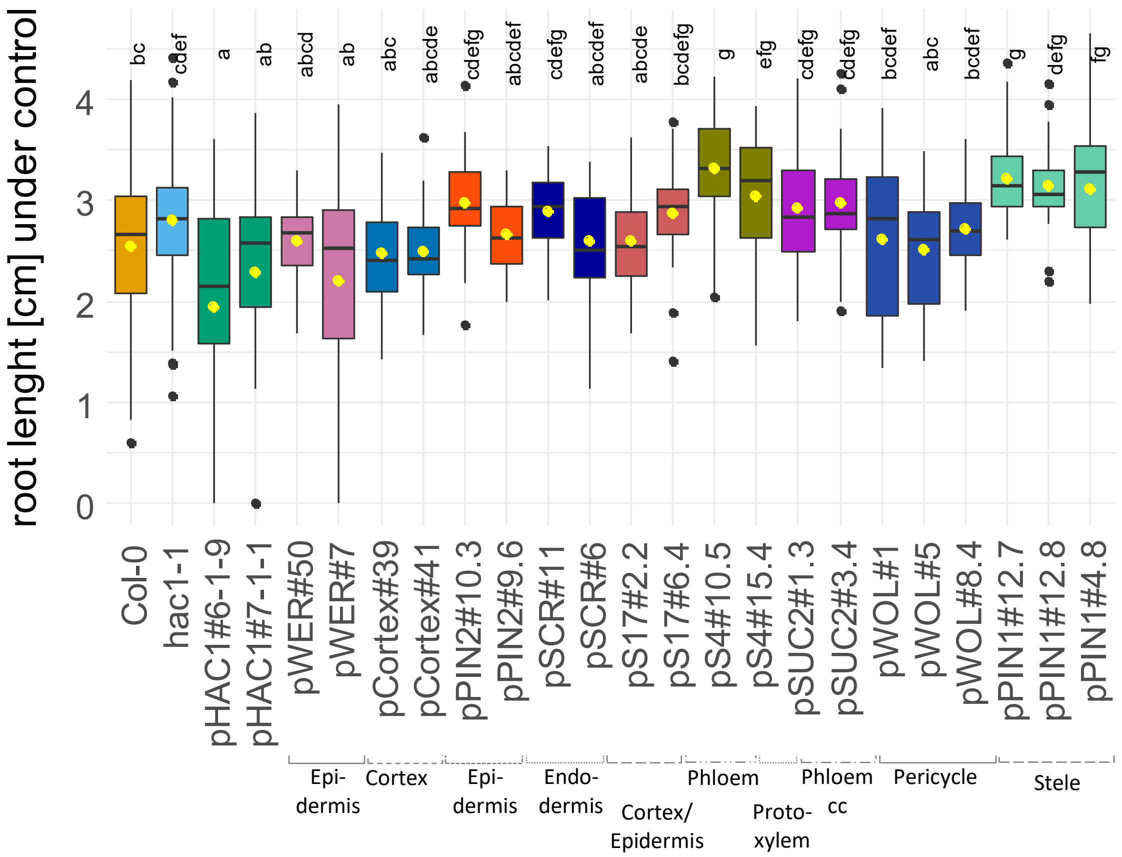

Fig. S2 Promotor activity. Growth under control conditions. The absolute root length under control conditions shows few differences between the lines. Box plots show the median +/- 1.58*IQR/sqrt(n) with outliers in black and the mean in yellow. Significant differences are shown with different letters above boxes, result of a One-way ANOVA with Post-hoc Tukey test. Bar plots show the mean ± standard deviation. n=20-169, Significant differences are shown with different letters above boxes, result of a One-way ANOVA with Post-hoc Tukey test.

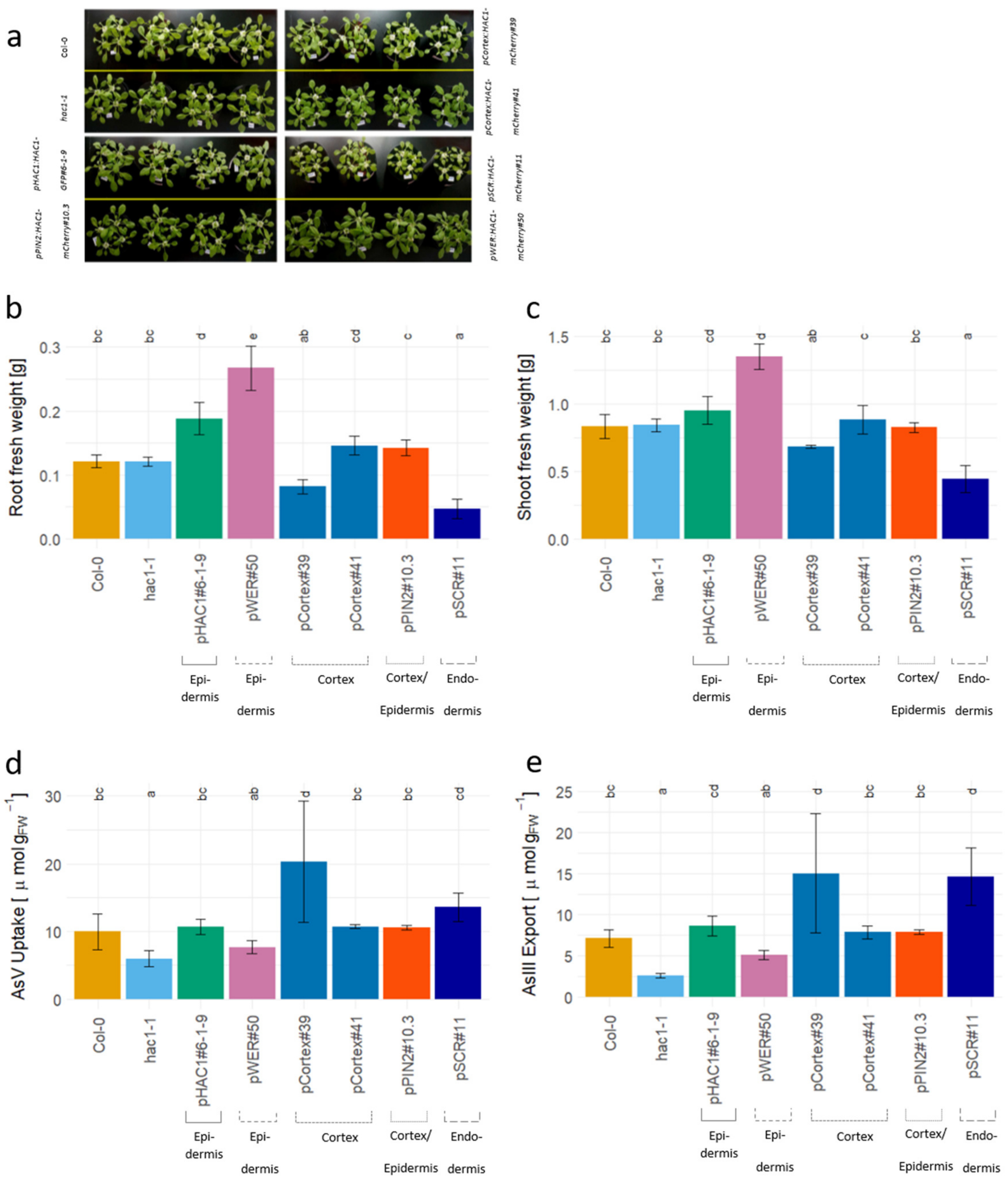

Fig. S3 As uptake and export in response to 200µM AsO_4Na_2H stress. Plants were grown hydroponically and (a) Images were taken to illustrate growth which was assessed after harvest by measuring (b) root and (c) shoot fresh weight. (d) As(V) uptake into the roots from the hydroponic medium was assessed as well as (e) As(III) export from the roots into the medium. Bar plots show the mean ± standard deviation. n=3 Significant differences are shown with different letters above boxes, result of a One-way ANOVA with Post-hoc Tukey test**.**

Table S1 Oligonucleotides used for sequencing the genetic constructs in bacteria and plants obtained in this work.

Oligo name	Sequence (5'-3')
**f-CORTEX sequencing**	GGATAGTTAAACCACGGACCAC
**r-CORTEX sequencing**	GTGGTCCGTGGTTTAACTATCC
**f-S4 sequencing**	GACCTCTTTCTTCTTCCCCTTTA
**r-S4 sequencing**	TAAAGGGGAAGAAGAAAGAGGTC
**f-SCR5 sequencing**	TACTAATATACAAGGGGACTAATGCT
**r-SCR5 sequencing**	GGAGATTGAAGGGTTGTTGGTC
**f-EXP7-1 sequencing**	CCCTGACATTCTCTCCCAAAG
**f-EXP7-2 sequencing**	AGCCACCACTACTGTCGTTGAA
**f-GL2 sequencing**	AGAGAGAGAGGGAGATGCGTATAG
**r-GL2 sequencing**	CTATACGCATCTCCCTCTCTCTCT
**f-PIN1-1 sequencing**	TGTAAGCAAACCAGAACCACCC
**f-PIN1-2 sequencing**	TCCACCACGCACCCATCA
**r-PIN1 sequencing**	TGATGGGTGCGTGGTGGA
**f-PIN2 sequencing**	TCAACCTTCGCCGTCTCAATA
**f-S17-1 sequencing**	CTTTGGAGAATGTTGTTTTTGTTG
**F-S17-2 sequencing**	AAGCGACCAAAAACTCTACTACAA
**r-S17-1 sequencing**	CAACAAAAACAACATTCTCCAAAG
**r-S17-2 sequencing**	CACCAATAGCCAATAGGACTCG
**f-SUC2-1 sequencing**	TACTATTATTCTCTGTCTTTATTCTCTTCAC
**f-SUC2-2 sequencing**	CAGTAAGATAGTTTGTGTGAAGGTAA
**r-SUC2-1 sequencing**	GTGAAGAGAATAAAGACAGAGAATAATAGTA
**f-WER sequencing**	CTCCGTCAAATAACTACTCAAAA
**r-WER sequencing**	CTTTTTGTTTCTTTGAATGATAGAC
**f-WOL sequencing**	GCCATGAAATTAAGGTATCCCA
**r-WOL sequencing**	TGGGATACCTTAATTTCATGGC
**f-promHAC1 sequencing**	GGTGTGTGGCCTCTAAAATCTC
**r-promHAC1 sequencing**	CGGGTCTCACGATTTATATATGC
**f-HAC1 ORF sequencing**	AGTTGTATGTATACATATTCTCTCC
**r-HAC1 ORF sequencing**	AGTTTTCCTTTGGCCTGAAC
**f-mCherry sequencing**	CCATCGTGGAACAGTACGAAC
**r-mCherry sequencing**	GATGGCCATGTTATCCTCCTCG
**f-eGFP sequencing**	CATGGTCCTGCTGGAGTTCGTG
**r-eGFP sequencing**	GTCCAGCTCGACCAGGATGGG
**f-M13 sequencing**	TGTAAAACGACGGCCAGT
**r-M13 sequencing**	GTTTTCCCAGTCACGAC

Table S2 Raw data for tissue quantification of root cross sections of Arabidospsis. Table contains information about source of the images, ImagJ results of the hand tranced area and the subsequent quantification of the contribution of each tissue to the overall root cross section area.

source	Image part	Tissue	Area in pixel	Area in % of total	Sum of Epidermis and Corte	Sum of Endodermis, Pericycle, Protoxylem, Phloem, Metaxylem and Procambium
**Hawker *et al*., 2004**	A	Epidermis	6954	0.41	0.79	0.19
**Hawker *et al*., 2004**	A	Cortex	6477	0.38		
**Hawker *et al*., 2004**	A	Endodermis	2362	0.14		
**Hawker *et al*., 2004**	A	Pericyle	451	0.03		
**Hawker *et al*., 2004**	A	Protoxylem	27	0.00		
**Hawker *et al*., 2004**	A	Phloem	94	0.01		
**Hawker *et al*., 2004**	A	Metaxylem	94	0.01		
**Hawker *et al*., 2004**	A	Procambium	207	0.01		
**Hawker *et al*., 2004**	A	Whole Root	16931	1.00		
**Hawker *et al*., 2004**	B	Epidermis	7469	0.47	0.82	0.19
**Hawker *et al*., 2004**	B	Cortex	5712	0.36		
**Hawker *et al*., 2004**	B	Endodermis	2154	0.13		
**Hawker *et al*., 2004**	B	Pericyle	419	0.03		
**Hawker *et al*., 2004**	B	Protoxylem	37	0.00		
**Hawker *et al*., 2004**	B	Phloem	66	0.00		
**Hawker *et al*., 2004**	B	Metaxylem	106	0.01		
**Hawker *et al*., 2004**	B	Procambium	264	0.02		
**Hawker *et al*., 2004**	B	Whole Root	16037	1.00		
**Dyson *et al*., 2014**	a	Epidermis	7468	0.45	0.76	0.21
**Dyson *et al*., 2014**	a	Cortex	5213	0.31		
**Dyson *et al*., 2014**	a	Endodermis	1893	0.11		
**Dyson *et al*., 2014**	a	Pericyle	784	0.05		
**Dyson *et al*., 2014**	a	Protoxylem	58	0.00		
**Dyson *et al*., 2014**	a	Phloem	231	0.01		
**Dyson *et al*., 2014**	a	Metaxylem	138	0.01		
**Dyson *et al*., 2014**	a	Procambium	400	0.02		
**Dyson *et al*., 2014**	a	Whole Root	16665	1.00		
**Sutka *et al*., 2011**	A	Epidermis	4913	0.39	0.79	0.19
**Sutka *et al*., 2011**	A	Cortex	5106	0.40		
**Sutka *et al*., 2011**	A	Endodermis	1134	0.09		
**Sutka *et al*., 2011**	A	Pericyle	408	0.03		

**Sutka *et al.*, 2011**	A	Protoxylem	54	0.00		
**Sutka *et al.*, 2011**	A	Phloem	164	0.01		
**Sutka *et al.*, 2011**	A	Metaxylem	223	0.02		
**Sutka *et al.*, 2011**	A	Procambium	419	0.03		
**Sutka *et al.*, 2011**	A	Whole Root	12750	1.00		
**Sutka *et al.*, 2011**	A	Epidermis	7170	0.34	0.76	0.25
**Sutka *et al.*, 2011**	A	Cortex	8895	0.42		
**Sutka *et al.*, 2011**	A	Endodermis	2546	0.12		
**Sutka *et al.*, 2011**	A	Pericyle	1277	0.06		
**Sutka *et al.*, 2011**	A	Protoxylem	70	0.00		
**Sutka *et al.*, 2011**	A	Phloem	363	0.02		
**Sutka *et al.*, 2011**	A	Metaxylem	236	0.01		
**Sutka *et al.*, 2011**	A	Procambium	845	0.04		
**Sutka *et al.*, 2011**	A	Whole Root	21017	1.00		
**Sutka *et al.*, 2011**	A	Epidermis	7615	0.29	0.70	0.26
**Sutka *et al.*, 2011**	A	Cortex	10518	0.41		
**Sutka *et al.*, 2011**	A	Endodermis	3122	0.12		
**Sutka *et al.*, 2011**	A	Pericyle	1266	0.05		
**Sutka *et al.*, 2011**	A	Protoxylem	119	0.00		
**Sutka *et al.*, 2011**	A	Phloem	576	0.02		
**Sutka *et al.*, 2011**	A	Metaxylem	465	0.02		
**Sutka *et al.*, 2011**	A	Procambium	1293	0.05		
**Sutka *et al.*, 2011**	A	Whole Root	25924	1.00		
**Sutka *et al.*, 2011**	A	Epidermis	7812	0.31	0.75	0.25
**Sutka *et al.*, 2011**	A	Cortex	10926	0.44		
**Sutka *et al.*, 2011**	A	Endodermis	3173	0.13		
**Sutka *et al.*, 2011**	A	Pericyle	1309	0.05		
**Sutka *et al.*, 2011**	A	Protoxylem	117	0.00		
**Sutka *et al.*, 2011**	A	Phloem	431	0.02		
**Sutka *et al.*, 2011**	A	Metaxylem	273	0.01		
**Sutka *et al.*, 2011**	A	Procambium	1003	0.04		
**Sutka *et al.*, 2011**	A	Whole Root	25085	1.00		
**Sutka *et al.*, 2011**	A	Epidermis	8173	0.33	0.72	0.23
**Sutka *et al.*, 2011**	A	Cortex	9768	0.39		
**Sutka *et al.*, 2011**	A	Endodermis	2766	0.11		
**Sutka *et al.*, 2011**	A	Pericyle	1044	0.04		
**Sutka *et al.*, 2011**	A	Protoxylem	66	0.00		
**Sutka *et al.*, 2011**	A	Phloem	410	0.02		
**Sutka *et al.*, 2011**	A	Metaxylem	301	0.01		
**Sutka *et al.*, 2011**	A	Procambium	1142	0.05		
**Sutka *et al.*, 2011**	A	Whole Root	25049	1.00		
**Sutka *et al.*, 2011**	B	Epidermis	11083	0.35	0.76	0.26
**Sutka *et al.*, 2011**	B	Cortex	13196	0.41		

**Sutka *et al*., 2011**	B	Endodermis	3472	0.11		
**Sutka *et al*., 2011**	B	Pericyle	1843	0.06		
**Sutka *et al*., 2011**	B	Protoxylem	124	0.00		
**Sutka *et al*., 2011**	B	Phloem	588	0.02		
**Sutka *et al*., 2011**	B	Metaxylem	373	0.01		
**Sutka *et al*., 2011**	B	Procambium	1863	0.06		
**Sutka *et al*., 2011**	B	Whole Root	31920	1.00		
**Sutka *et al*., 2011**	B	Epidermis	6510	0.34	0.77	0.17
**Sutka *et al*., 2011**	B	Cortex	7992	0.42		
**Sutka *et al*., 2011**	B	Endodermis	1561	0.08		
**Sutka *et al*., 2011**	B	Pericyle	913	0.05		
**Sutka *et al*., 2011**	B	Protoxylem	40	0.00		
**Sutka *et al*., 2011**	B	Phloem	183	0.01		
**Sutka *et al*., 2011**	B	Metaxylem	102	0.01		
**Sutka *et al*., 2011**	B	Procambium	479	0.03		
**Sutka *et al*., 2011**	B	Whole Root	18884	1.00		
**Sutka *et al*., 2011**	B	Epidermis	7505	0.36	0.73	0.23
**Sutka *et al*., 2011**	B	Cortex	7769	0.37		
**Sutka *et al*., 2011**	B	Endodermis	2228	0.11		
**Sutka *et al*., 2011**	B	Pericyle	1001	0.05		
**Sutka *et al*., 2011**	B	Protoxylem	169	0.01		
**Sutka *et al*., 2011**	B	Phloem	300	0.01		
**Sutka *et al*., 2011**	B	Metaxylem	296	0.01		
**Sutka *et al*., 2011**	B	Procambium	737	0.04		
**Sutka *et al*., 2011**	B	Whole Root	20822	1.00		
**Sutka *et al*., 2011**	B	Epidermis	6452	0.38	0.76	0.23
**Sutka *et al*., 2011**	B	Cortex	6570	0.38		
**Sutka *et al*., 2011**	B	Endodermis	1727	0.10		
**Sutka *et al*., 2011**	B	Pericyle	912	0.05		
**Sutka *et al*., 2011**	B	Protoxylem	72	0.00		
**Sutka *et al*., 2011**	B	Phloem	356	0.02		
**Sutka *et al*., 2011**	B	Metaxylem	191	0.01		
**Sutka *et al*., 2011**	B	Procambium	596	0.03		
**Sutka *et al*., 2011**	B	Whole Root	17107	1.00		
**Sutka *et al*., 2011**	B	Epidermis	8433	0.29	0.70	0.28
**Sutka *et al*., 2011**	B	Cortex	12139	0.41		
**Sutka *et al*., 2011**	B	Endodermis	3697	0.13		
**Sutka *et al*., 2011**	B	Pericyle	1791	0.06		
**Sutka *et al*., 2011**	B	Protoxylem	140	0.00		
**Sutka *et al*., 2011**	B	Phloem	621	0.02		
**Sutka *et al*., 2011**	B	Metaxylem	283	0.01		
**Sutka *et al*., 2011**	B	Procambium	1795	0.06		
**Sutka *et al*., 2011**	B	Whole Root	29304	1.00		
